# CA-125 Levels Are Predictive of Survival in Low-Grade Serous Ovarian Cancer—A Multicenter Analysis

**DOI:** 10.3390/cancers14081954

**Published:** 2022-04-13

**Authors:** Christoph Wohlmuth, Vladimir Djedovic, Susanne K. Kjaer, Allan Jensen, Rosalind Glasspool, Patricia Roxburgh, Anna DeFazio, Sharon E. Johnatty, Penelope M. Webb, Francesmary Modugno, Diether Lambrechts, Joellen M. Schildkraut, Andrew Berchuck, Liv Cecilie Vestrheim Thomsen, Line Bjorge, Estrid Høgdall, Claus K. Høgdall, Ellen L. Goode, Stacey J. Winham, Keitaro Matsuo, Beth Y. Karlan, Jenny Lester, Marc T. Goodman, Pamela J. Thompson, Tanja Pejovic, Marjorie J. Riggan, Katherine Lajkosz, Alicia Tone, Taymaa May

**Affiliations:** 1 Division of Gynecologic Oncology, Princess Margaret Hospital, University Health Network, Toronto, ON M5G 2M9, Canada; c.wohlmuth@salk.at (C.W.); vdjed024@uottawa.ca (V.D.); atone@ovariancanada.org (A.T.); 2 Department of Obstetrics and Gynecology, Paracelsus Medical University, 5020 Salzburg, Austria; 3 Department of Internal Medicine, University of Ottawa, Ottawa, ON K1H 8M5, Canada; 4 Department of Lifestyle, Reproduction and Cancer, Danish Cancer Society Research Center, DK-2100 Copenhagen, Denmark; susanne@cancer.dk (S.K.K.); allan@cancer.dk (A.J.); 5 Department of Gynaecology, Rigshospitalet, University of Copenhagen, DK-2100 Copenhagen, Denmark; claus.hogdall@regionh.dk; 6 Beatson West of Scotland Cancer Centre, University of Glasgow, Glasgow G12 0YN, UK; ros.glasspool@ggc.scot.nhs.uk (R.G.); patricia.roxburgh@glasgow.ac.uk (P.R.); 7 Institute of Cancer Sciences, University of Glasgow, Glasgow G61 1QH, UK; 8 Centre for Cancer Research, The Westmead Institute for Medical Research, Sydney, NSW 2145, Australia; anna.defazio@sydney.edu.au; 9 Department of Gynaecological Oncology, Westmead Hospital, Sydney, NSW 2145, Australia; 10 The Daffodil Centre, a Joint Venture with Cancer Council NSW, The Daffodil Centre, The University of Sydney, Sydney, NSW 2145, Australia; 11 Cancer Genetics Laboratory, Research Division, Peter MacCallum Cancer Center, Melbourne, VIC 3000, Australia; 12 Department of Genetics and Computational Biology, QIMR Berghofer Medical Research Institute, Brisbane, QLD 4006, Australia; sharon.johnatty@qimrberghofer.edu.au; 13 Population Health Department, QIMR Berghofer Medical Research Institute, Brisbane, QLD 4006, Australia; penny.webb@qimrberghofer.edu.au; 14 Women’s Cancer Research Center, Magee-Womens Research Institute and Hillman Cancer Center, Pittsburgh, PA 15213, USA; modugnof@mwri.magee.edu; 15 Department of Obstetrics, Gynecology and Reproductive Sciences, University of Pittsburgh School of Medicine, Pittsburgh, PA 15213, USA; 16 Laboratory for Translational Genetics, Department of Human Genetics, KU Leuven, 3000 Leuven, Belgium; diether.lambrechts@kuleuven.vib.be; 17 VIB Center for Cancer Biology, VIB, 3000 Leuven, Belgium; 18 Department of Epidemiology, Rollins School of Public Health, Emory University, Atlanta, GA 30322, USA; jmschil@emory.edu; 19 Department of Gynecologic Oncology, Duke University Hospital, Durham, NC 27710, USA; berch001@mc.duke.edu (A.B.); marjorie.riggan@duke.edu (M.J.R.); 20 Department of Obstetrics and Gynecology, Haukeland University Hospital, 5021 Bergen, Norway; liv.vestrheim@uib.no (L.C.V.T.); line.bjorge@uib.no (L.B.); 21 Centre for Cancer Biomarkers CCBIO, Department of Clinical Science, University of Bergen, 5020 Bergen, Norway; 22 Department of Pathology, Herlev Hospital, University of Copenhagen, DK-2100 Copenhagen, Denmark; estrid.hoegdall@regionh.dk; 23 Division of Epidemiology, Department of Quantitative Health Sciences, Mayo Clinic, Rochester, MN 55905, USA; egoode@mayo.edu; 24 Division of Computational Biology, Department of Quantitative Health Sciences, Mayo Clinic, Rochester, MN 55905, USA; winham.stacey@mayo.edu; 25 Division of Cancer Epidemiology and Prevention, Aichi Cancer Center Research Institute, Nagoya 464-8681, Japan; kmatsuo@aichi-cc.jp; 26 Division of Cancer Epidemiology, Nagoya University Graduate School of Medicine, Nagoya 466-8550, Japan; 27 Department of Obstetrics and Gynecology, David Geffen School of Medicine, University of California at Los Angeles, Los Angeles, CA 90095, USA; bkarlan@mednet.ucla.edu (B.Y.K.); jlester@mednet.ucla.edu (J.L.); 28 Cancer Prevention and Control Program, Cedars-Sinai Cancer, Cedars-Sinai Medical Center, Los Angeles, CA 90048, USA; marc.goodman@cshs.org; 29 Samuel Oschin Comprehensive Cancer Institute, Cancer Prevention and Genetics Program, Cedars-Sinai Medical Center, Los Angeles, CA 90048, USA; pam@cc.hawaii.edu; 30 Department of Obstetrics and Gynecology, Oregon Health & Science University, Portland, OR 97239, USA; pejovict@ohsu.edu; 31 Knight Cancer Institute, Oregon Health & Science University, Portland, OR 97239, USA; 32 Biostatistics, Princess Margaret Cancer Centre, University of Toronto, Toronto, ON M5G 2C1, Canada; katherine.lajkosz@uhnresearch.ca

**Keywords:** ovarian cancer, low-grade serous cancer, CA-125, survival

## Abstract

**Simple Summary:**

Low-grade serous cancer (LGSC) accounts for approximately 5% of all ovarian cancers. It is characterized by its high resistance to chemotherapy. Cytoreductive surgery, therefore, is the primary treatment modality for this disease and previous studies have shown that complete removal of all visible tumor tissue should be achieved. In this study, 176 women with LGSC were included and most of them had advanced disease stages, where the disease had already spread. CA-125 is a biomarker that has been previously studied in ovarian cancer. We have found that CA-125 level following treatment of LGSC is an important and independent prognostic factor for progression-free and overall survival. It may be a better surrogate for the true amount of residual disease following treatment compared to the gross estimation of visible residual disease during surgery.

**Abstract:**

Objective: Studies on low-grade serous ovarian cancer (LGSC) are limited by a low number of cases. The aim of this study was to define the prognostic significance of age, stage, and CA-125 levels on survival in a multi-institutional cohort of women with pathologically confirmed LGSC. Methods: Women with LGSC were identified from the collaborative Ovarian Cancer Association Consortium (OCAC). Cases of newly diagnosed primary LGSC were included if peri-operative CA-125 levels were available. Age at diagnosis, FIGO stage, pre- and post-treatment CA-125 levels, residual disease, adjuvant chemotherapy, disease recurrence, and vital status were collected by the participating institutions. Progression-free (PFS) and overall survival (OS) were calculated. Multivariable (MVA) Cox proportional hazard models were used and hazard ratios (HR) calculated. Results: A total of 176 women with LGSC were included in this study; 82% had stage III/IV disease. The median PFS was 2.3 years and the median OS was 6.4 years. Age at diagnosis was not significantly associated with worse PFS (*p* = 0.23) or OS (*p* = 0.3) (HR per year: 0.99; 95%CI, 0.96–1.01 and 0.98; 95%CI 0.95–1.01). FIGO stage III/IV was independently associated with PFS (HR 4.26, 95%CI 1.43–12.73) and OS (HR 1.69, 95%CI 0.56–5.05). Elevated CA-125 (≥35 U/mL) at diagnosis was not significantly associated with worse PFS (*p* = 0.87) or OS (*p* = 0.78) in MVA. Elevated CA-125 (≥35 U/mL) after completion of primary treatment was independently associated with worse PFS (HR 2.81, 95%CI 1.36–5.81) and OS (HR 6.62, 95%CI 2.45–17.92). In the MVA, residual disease was independently associated with PFS (0.022), but not OS (0.85). Conclusion: Advanced LGSC was associated with poor long-term prognosis. FIGO stage and abnormal post-treatment CA-125 level are key prognostic factors inversely associated with PFS and OS. Highlights: 1. Through a multi-center collaborative effort, data from 176 women with low-grade serous ovarian cancer were analyzed. 2. Although low-grade serous ovarian cancer is often considered indolent, the progression-free and overall survival are poor. 3. Elevated post-treatment CA-125 levels are independently associated with poor survival.

## 1. Introduction

Epithelial ovarian cancer (EOC) is the leading cause of death from gynecologic malignancies in the United States, Canada, and Europe [1,2,3]. Serous carcinoma is the most common subtype of epithelial ovarian malignancies. A two-tier classification system distinguishes high-grade (HGSC) from low-grade serous ovarian carcinoma (LGSC) based on the degree of nuclear atypia [4,5]. Recent evidence suggests that the more common HGSC clearly differs from the less common LGSC with regard to cells of origin, molecular pathogenesis, clinical behavior, and pathologic characteristics. Thus, LGSC and HGSC are considered two distinct malignancies [6,7,8].

LGSC accounts for approximately 5% of serous ovarian malignancies and may arise from serous low malignant potential tumors of the ovary or de novo from the ovary or peritoneum [9,10]. While the tumor typically evolves slower, existing evidence suggests limited benefit of cytotoxic therapy given the relatively high resistance of LGSC to platinum-based chemotherapy [11,12]. A retrospective study from the German AGO registry found an objective response rate of 23% for women with suboptimal cytoreduction of advanced LGSC given adjuvant chemotherapy, as compared to a 90% response rate in a similar population of patients with HGSC [13]. Moreover, objective response rates of <5% have been reported for LGSC in the neoadjuvant and recurrent setting [14,15]. Surgery is therefore considered the cornerstone of treatment for LGSC; however, novel systemic and targeted treatment modalities are needed in conjunction with surgery given the poor chemosensitivity of these tumors.

Due to the indolent nature of LGSC, patients may experience either a protracted clinical course, or in the case of treatment-refractory disease, a more rapid progression where palliative care is typically required soon after treatment initiation [16]. The identification of prognostic factors can aid in clinical decision-making with regard to treatment [17]. Therefore, recent efforts have been directed towards identifying and contextualizing known, and novel, prognostic factors into clinical practice. For EOC in general, tumor stage and grade, physical status at time of diagnosis, body mass index (BMI), extent of surgical cytoreduction, heredity, immunological factors, and molecular biomarkers have been identified [18].

The aim of this study was to define the prognostic significance of age, stage, residual disease, and pre- and post-treatment CA-125 levels in terms of survival in a large multi-institutional cohort of women with pathologically confirmed LGSC.

## 2. Methods

### 2.1. Patient Population

Women with LGSC were identified from the international collaborative Ovarian Cancer Association Consortium (OCAC). The OCAC consortium was founded in 2005 to combine data from individual studies on ovarian cancer [19]. Clinical data were collected at each participating site and entered into a universal de-identified clinical database. Only women with newly diagnosed, primary LGSC who underwent primary surgical resection were included in this analysis. A pathology report by a specialized gynecologic oncology pathologist confirming the diagnosis of LGSC, the documented date of birth, and pre-operative, diagnostic CA-125 levels, prior to the commencement of any therapy and following completion of primary treatment, were also required.

Patient demographics and clinical data included: age at diagnosis, International Federation of Obstetrics and Gynecology (FIGO) stage, CA-125 level at diagnosis, CA-125 level after treatment completion, residual disease-status following primary cytoreductive surgery (no macroscopic disease, macroscopic disease ≤ 1 cm, and macroscopic disease > 1 cm), first-line adjuvant chemotherapy, disease recurrence, and vital status. For CA-125, a cut-off of 35 was used as generally accepted. In addition, secondary analyses were performed comparing patients with CA-125 levels < 35, 35–500 and >500 to explore the difference between moderately increased and very high levels of CA-125. Research ethics board approval was obtained at the participating sites.

### 2.2. Statistical Analyses

Descriptive statistics were used to report demographic data. Chi square tests were used to compare categorical data. Progression-free survival (PFS) was calculated from the date of diagnosis to the date of first progression or the date of death. Overall survival (OS) was calculated from the date of diagnosis to the date of death. Patients who remained alive were censored on the date of last follow-up. PFS and OS were estimated using the Kaplan–Meier method with log-rank tests to examine survival differences. Univariable (UVA) and multivariable (MVA) Cox proportional hazards models were fit to assess the association of age, FIGO stage, residual disease, and CA-125 with PFS and OS. The proportional hazards assumption for the multivariable model was verified; the models were stratified by study site; and *p*-values < 0.05 were considered statistically significant. All tests were two-sided. Statistical analyses were performed using R version 3.6.0, www.r-project.org accessed on 1 April 2022 and SPSS Version 26, IBM, Armonk, NY, USA.

## 3. Results

### 3.1. Patient Population

A total of 176 women with LGSC from 15 cancer centers within the OCAC collaborative network were included in this study. The number of patients included from the individual study cohorts is shown in Supplementary Table S1. Patient demographics and clinical characteristics are shown in Table 1. The median age at diagnosis was 54 years (range 18–80 years). FIGO stage was reported in 172 patients (98%). The majority of patients were diagnosed with advanced disease, with the following disease distribution noted: 14 patients (8%) had stage I disease, 17 (10%) stage II, 125 (73%) stage III, and 16 (9%) stage IV disease. A total of 114 patients (65%) had information on cytoreductive surgical outcome. Complete cytoreduction to no macroscopic residual disease was achieved in 57% of patients, optimal cytoreduction to macroscopic residual disease ≤ 1 cm was achieved in 26% of patients, and suboptimal cytoreduction with macroscopic disease > 1 cm was performed in 17% of cases. There was a statistical trend towards higher rates of suboptimal cytoreduction in older women, where 57% of women younger than 54 years had complete cytoreduction to no macroscopic residual disease, 33% had optimal cytoreduction to macroscopic residual disease ≤ 1 cm, and 10% had suboptimal cytoreduction with macroscopic disease > 1 cm. In women older than 54 years, 57% had complete cytoreduction to no macroscopic residual disease, 20% had optimal cytoreduction to macroscopic residual disease ≤ 1 cm, and 23% had suboptimal cytoreduction with macroscopic disease > 1 cm (*p* = 0.096).

**Figure 1:**
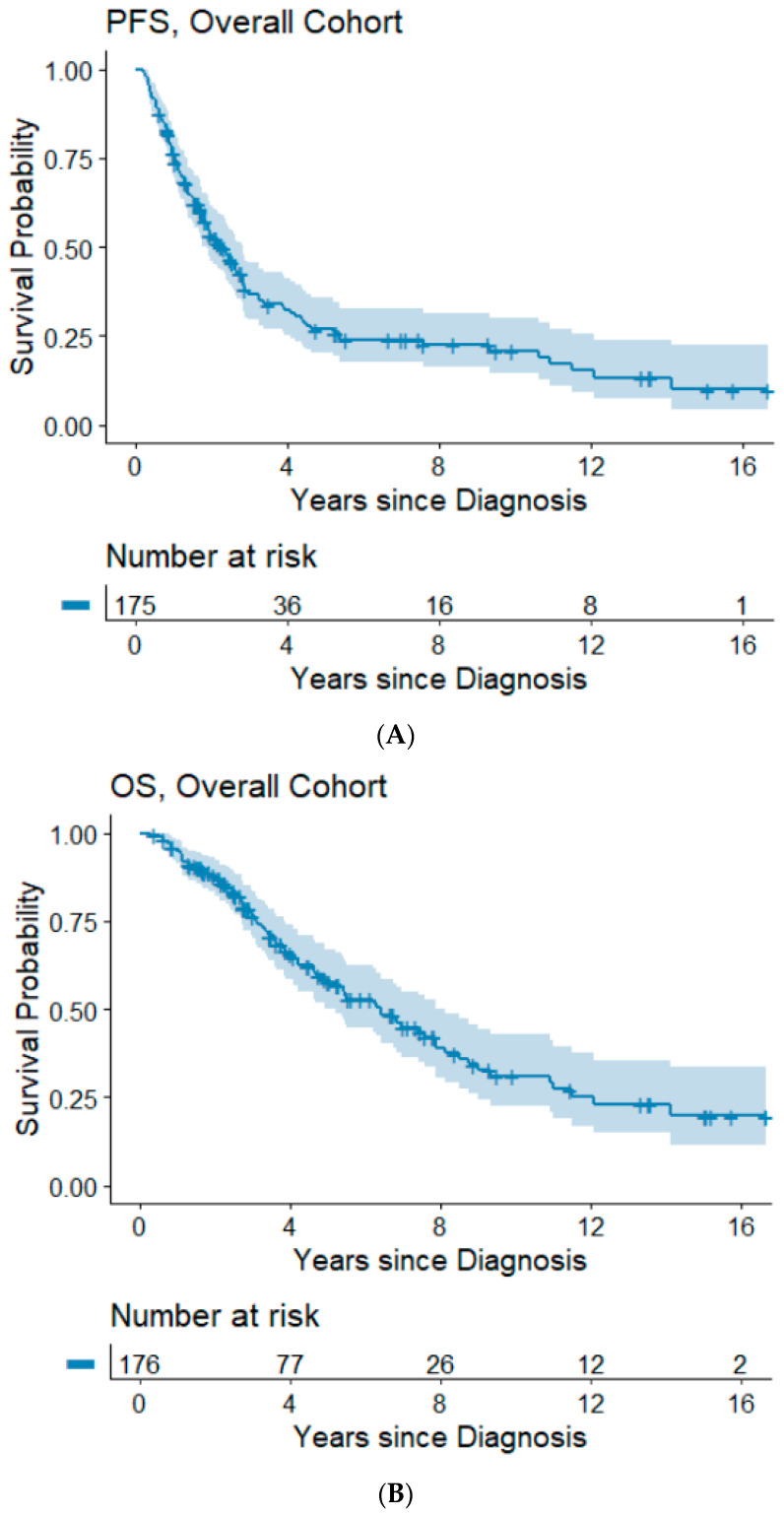
(**A**,**B**) Progression-free and overall survival of the overall cohort. Abbreviations: OS, overall survival; PFS, progression-free survival.

The median follow-up time was 3.5 years (range: 0.3–16.7 years). Information on progression/recurrence status was available for 175 patients, of which 120 (68%) experienced disease progression. The median time to progression was 2.3 years and the 5- and 10-year PFS rates were 26.8% and 20.9%. In our cohort, 85 (48%) patients had died. The median OS was 6.4 years and the 5- and 10-year OS rates were 57.8% and 31.1%, respectively. Kaplan–Meier survival plots for PFS and OS are shown in Figure 1A,B.

### 3.2. The Impact of Clinical Parameters on Survival

**Figure 2:**
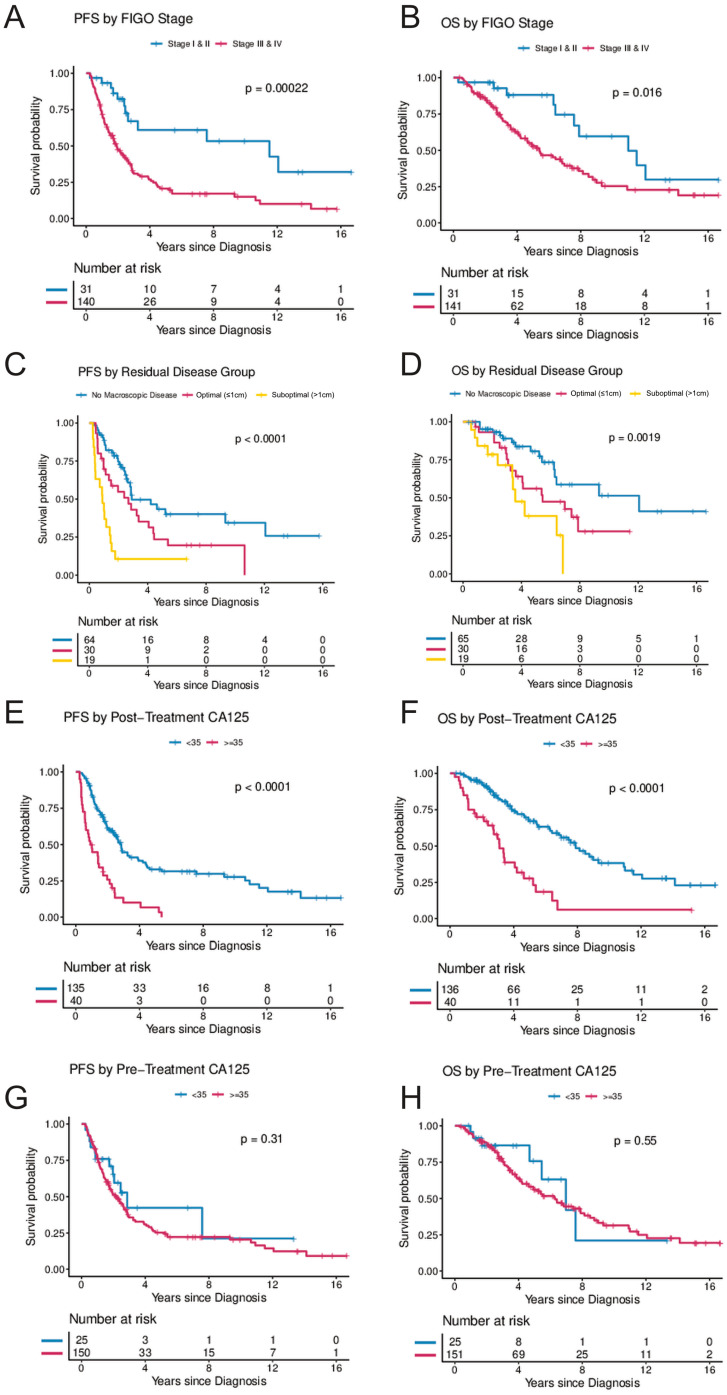
(**A**–**H**) Progression-free and overall survival by FIGO stage, residual disease, and post-treatment CA-125 and pre-treatment CA-125. (**A**,**B**) illustrate progression-free and overall survival by FIGO stage (Stages I and II combined vs. Stages III and IV). (**C**,**D**) illustrate progression-free and overall survival by residual disease following cytoreductive surgery (no visible macroscopic disease vs. macroscopic disease ≤ 1 cm vs. macroscopic disease > 1 cm). (**E**,**F**) illustrate progression-free and overall survival by CA-125 levels following treatment (<35 vs. ≥35) and (**G**,**H**) illustrate progression-free and overall survival by CA-125 levels at diagnosis (<35 vs. ≥35). The Kaplan–Meier method with log-rank tests was used to examine survival differences. *p*-values < 0.05 were considered statistically significant. Individuals at risk are shown in the legends. Abbreviations: OS, overall survival; PFS, progression-free survival.

FIGO stage at diagnosis was significantly associated with PFS and OS (Table 2 and Figure 2A,B) in the UVA, and with PFS in the MVA. Of women with stage III/IV disease, 70.9% experienced disease progression during the observation period and the median time to progression was 1.7 years. In the MVA, stage III and IV were associated with a hazard ratio (HR) of 4.26 (95%CI, 1.43–12.73) for progression, and 1.69 (95%CI, 0.56–5.05) for death, respectively.

Residual disease after surgical cytoreduction was significantly associated with PFS. Suboptimal cytoreduction with macroscopic residual disease > 1 cm compared to complete cytoreduction with no macroscopic residual disease was associated with an HR of 2.93 (95%CI, 1.33–6.47) for progression and 1.06 (95%CI, 0.4–2.81) for death, respectively (Table 2 and Figure 2C,D).

**Figure 3:**
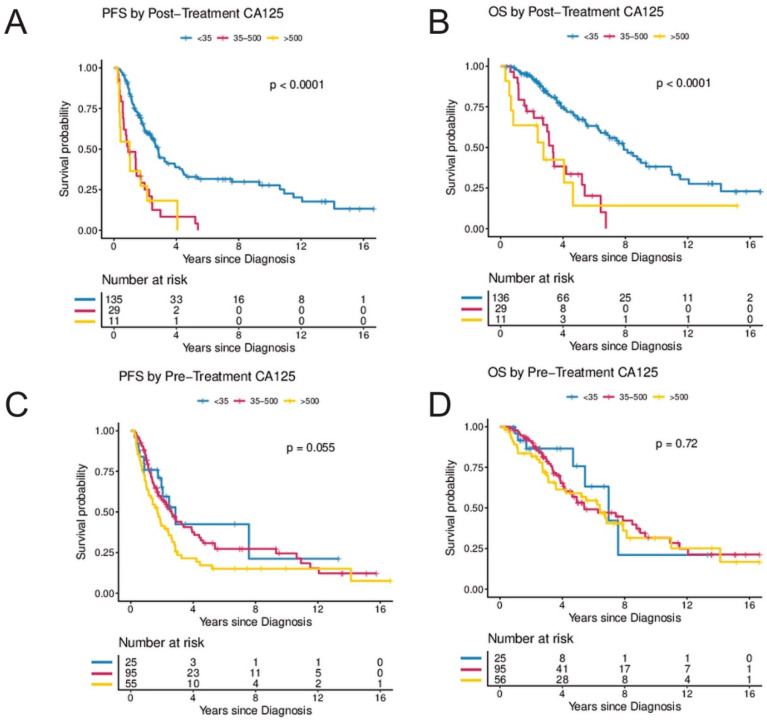
(**A**–**D**) Secondary analysis of post-treatment and pre-treatment CA-125 by CA-125 level. (**A**,**B**) illustrate progression-free and overall survival by CA-125 levels following treatment (<35 vs. 35–500 vs. >500) and (**C**,**D**) illustrate progression-free and overall survival by CA-125 levels at diagnosis (<35 vs. 35–500 vs. >500). The Kaplan–Meier method with log-rank tests was used to examine survival differences. *p*-values < 0.05 were considered statistically significant. Individuals at risk are shown in the legends. Abbreviations: OS, overall survival; PFS, progression-free survival.

Elevated CA-125 levels at diagnosis prior to surgery were not associated with PFS or OS when compared to patients with normal CA-125 levels at diagnosis (Figure 2G,H). In contrast, women with abnormal post-treatment CA-125 (≥35 U/mL) had a significantly higher risk for progression and death. In the UVA, an elevated post-treatment CA-125 level was associated with an HR of 3.45 (95%CI, 2.23–5.33) for progression and 3.58 (95%CI, 2.19–5.87) for death, respectively. In the MVA (when controlling for age, stage, and residual disease), CA-125, following completion of treatment was independently associated with risk of progression (HR 2.93; 95%CI, 1.36–5.81; *p* = 0.005) and death (HR 6.62; 95%CI, 2.45–17.92; *p* < 0.001) (Table 2 and Figure 2E,F). In a secondary analysis, the range of CA-125 values at diagnosis were analyzed by comparing survival in women with baseline CA-125 < 35 to women with baseline CA-125 levels between 35 and 500 and to women with baseline CA-125 levels > 500. There was a trend towards improved PFS in women with pre-treatment CA-125 < 35 (*p* = 0.055) but no statistically significant difference in OS (*p* = 0.72) (Figure 3C,D). In contrast, women with normal post-treatment CA-125 < 35 had significantly better PFS (*p* < 0.0001) and OS (*p* < 0.0001) when compared to those with post-treatment CA-125 between 35–500 and those with post-treatment CA-125 > 500 (Figure 3A,B).

## 4. Discussion

In this study, the prognostic significance of age, FIGO stage, residual disease, and pre- and post-treatment CA-125 levels were analyzed from a comprehensive multi-center cohort of 176 women with newly diagnosed and pathologically confirmed LGSC. Age at diagnosis was not an independent predictor for progression or death. FIGO stage was found to be an independent prognostic factor for disease progression. Importantly, LGSC is relatively chemoresistant and is primarily treated with surgical cytoreduction. We have observed that women with suboptimal cytoreduction have a significantly worse prognosis, which is in agreement with previous findings from a subgroup analysis of GOG-182 study which found that women with LGSC who had suboptimal cytoreduction with >1 cm residual disease have a worse outcome [8]. Women in this cohort with complete cytoreduction with no macroscopic residual disease had improved outcomes, followed by women with optimal cytoreduction with macroscopic residual disease ≤ 1 cm as compared to women with suboptimal cytoreduction with macroscopic residual disease > 1 cm. Unlike HGSC, medical treatment options for LGSC are limited. A study by the AGO found a limited benefit to systemic chemotherapy with poor response rates in LGSC when compared to HGSC [13]. In a recent phase 2/3 clinical trial assessing trametinib versus standard of care regimens (intravenous paclitaxel 80 mg/m^2^ by body surface area on days 1, 8, and 15 of every 28-day cycle; intravenous pegylated liposomal doxorubicin 40–50 mg/m^2^ by body surface area once every 4 weeks; intravenous topotecan 4 mg/m^2^ by body surface area on days 1, 8, and 15 of every 28-day cycle; oral letrozole 2·5 mg once daily; or oral tamoxifen 20 mg twice daily), trametinib was associated with improved median progression-free survival (13.0 months (95%CI 9.9–15.0) compared with 7.2 months (5.6–9.9) in the standard-of-care group (hazard ratio 0.48 [95%CI 0.36–0.64]; *p* < 0.0001) [20]. However, surgery remains the primary treatment modality for LGSC. Therefore, careful evaluation and referral to a gynecologic oncologist for consideration of primary surgery with the aim for complete or optimal surgical cytoreduction is paramount for women with newly diagnosed LGSC including those presenting at older age.

CA-125 is a large membrane glycoprotein encoded by the gene *MUC16* that is overexpressed in 85% of serous ovarian cancers [21,22,23]. Interestingly, in our cohort, elevated CA-125 at diagnosis was not associated with worse PFS or OS. However, we found a significant association of post-treatment CA-125 levels with the risk of progression and death in this cohort. Previous studies have shown that the nadir of CA-125 levels are associated with PFS and OS in high-grade serous ovarian cancer [24,25]. In the MVA, after controlling for age and FIGO stage, an elevated post-treatment CA-125 level remained an independent risk factor for disease progression and death. This may aid in identifying women at higher risk of relapse after completing primary treatment and may guide additional treatments, monitoring and follow-up. Importantly, the value of post-treatment CA-125 appears to be a better predictor of OS than residual disease. One possible explanation is that the post-treatment CA-125 level may be a better indicator of the true amount of residual disease following treatment, completion of surgery, and systemic therapy than gross estimation of visible residual disease.

Our study provides data from a well characterized, international multi-center cohort of women with LGSC. All original pathology reports were generated by subspecialized gynecologic pathologists. The strengths of this work include the relatively large number of patients, especially in this rare disease. The study is limited by its retrospective nature and the lack of central pathology review.

## 5. Conclusions

In summary, advanced LGSC was associated with poor prognosis and low long-term survival in this international cohort of patients. FIGO stage at diagnosis and the amount of residual disease following surgical cytoreduction are important prognostic factors. Age and elevated CA-125 levels at presentation were not associated with worse survival outcomes. Abnormal CA-125 level after completing treatment was an independent predictor of recurrence and worse survival.

## Figures and Tables

**Table 1:** Demographic and clinical characteristics.

Parameter	
Age at diagnosis, years	
Mean ± SD	53.8 ± 13.5
Median (range)	54 (18–80)
Ethnicity	
White, *n* (%)	112 (64%)
Black, *n* (%)	3 (2%)
Asian, *n* (%)	2 (1%)
Other, *n* (%)	3 (2%)
not reported, *n*	56 (32%)
FIGO stage	
Stage I, *n* (%)	14 (8%)
Stage II, *n* (%)	17 (10%)
Stage III, *n* (%)	125 (73%)
Stage IV, *n* (%)	16 (9%)
not specified, *n*	4
Residual Disease	
No macroscopic disease	65 (57%)
Optimal, ≤1 cm	30 (26%)
Suboptimal, >1 cm	19 (17%)
not reported, *n*	62
Pre-Treatment CA-125	
<35	25 (14%)
≥35	151 (86%)
Post-Treatment CA-125	
<35	136 (77%)
≥35	40 (23%)
Total Follow Up, Years	
Mean ± SD	4.7 ± 3.7

Abbreviations: *n*, number; SD, standard deviation.

**Table 2:** Univariate and multivariate cox proportional hazard model for progression-free and overall survival.

		PFS				OS			
Parameter		Univariate		Multivariate		Univariate		Multivariate	
		HR (95%CI)	*p*	HR (95%CI)	*p*	HR (95%CI)	*p*	HR (95%CI)	*p*
Age									
Age, continuous		1.01 (0.99–1.02)	0.31	0.99 (0.96–1.01)	0.23	1.01 (0.99–1.03)	0.25	0.98 (0.95–1.01)	0.3
FIGO stage			<0.001		0.009		0.026		0.35
Stage I/II		Reference		Reference		Reference		Reference	
Stage III/IV		3.77 (1.94–7.35)	<0.001	4.26 (1.43–12.73)	0.009	2.16 (1.09–4.27)	0.026	1.69 (0.56–5.05)	0.35
Residual Disease			<0.001		0.022		0.043		0.85
No macroscopic		Reference		Reference		Reference		Reference	
Optimal, ≤1 cm		1.77 (0.78–4.01)	0.17	1.26 (0.54–2.92)	0.59	2.14 (0.82–5.59)	0.12	1.33 (0.50–3.55)	0.57
Suboptimal, >1 cm		4.98 (2.38–10.44)	<0.001	2.93 (1.33–6.47)	0.008	2.84 (1.24–6.51)	0.014	1.06 (0.4–2.81)	0.91
PreTx CA-125			0.28		0.87		0.37		0.78
<35		Reference		Reference		Reference		Reference	
≥35		1.43 (0.75–2.74)	0.28	1.07 (0.46–2.52)	0.87	1.44 (0.65–3.22)	0.37	1.17 (0.39–3.45)	0.78
PostTx CA-125			<0.001		0.005		<0.001		<0.001
<35		Reference		Reference		Reference		Reference	
≥35		3.45 (2.23–5.33)	0.003	2.81 (1.36–5.81)	0.005	3.58 (2.19–5.87)	<0.001	6.62 (2.45–17.92)	<0.001

Abbreviations: CI, confidence interval; HR hazard ratio; OS, overall survival; PFS, progression-free survival; PostTx CA-125, CA-125 level within 3 months of completion of first-line chemotherapy and prior to commencement of maintenance therapy; PreTx CA-125, CA-125 level before treatment initiation (primary surgery or neoadjuvant chemotherapy).

## Data Availability

Data are available on request from the corresponding author.

## Supplementary Materials

The following supporting information can be downloaded at: https://www.mdpi.com/article/10.3390/cancers14081954/s1, Table S1: OCAC study sites with the number of included cases (*n* = 176).
