# Supplementary material for: CA-125 Levels Are Predictive of Survival in Low-Grade Serous Ovarian Cancer—A Multicenter Analysis
Source: Cancers (Basel). 2022 Apr 13;14(8):1954. doi: 10.3390/cancers14081954 (PMC9024456; doi:10.3390/cancers14081954)
Supplement: Supplementary file 1 [file cancers-14-01954-s001.zip › cancers-1662428-supplementary.pdf]

Table S1. OCAC study sites with the number of included cases (n = 176).

OCAC Site ID	Cases	Study Name (Acronym, Country)	Study Type
AUS	26	Australia Ovarian Cancer Study & Australia Cancer Study (Ovarian Cancer) (AOCS/ACS; Australia)	Case–control
BEL	17	Belgium Ovarian Cancer Study (BOCS; Belgium)	Case–control
JPN	2	Hospital-based Epidemiologic Research Program at Aichi Cancer Center (HERPACC; Japan)	Case–control
MAL	49	Danish Malignant Ovarian Tumor Study (MALOVA; Denmark)	Case–control
MAY	2	Mayo Clinic Ovarian Cancer Case Control Study (USA)	Case–control
NCO	11	North Carolina Ovarian Cancer Study (NCOCS; USA)	Case–control
NOR	7	University of Bergen, Haukeland University Hospital (Norway)	Case–control
OPL	13	Ovarian Cancer Prognosis and Lifestyle Study (OPAL; Australia)	Case-only
ORE	1	Oregon Ovarian Cancer Registry (OHSU- OOCR; USA)	Case-only
SRO	31	Scottish Randomised Trial in Ovarian Cancer (SCOTROC; UK)	Case-only (trial)
UHN	17	Princess Margaret Cancer Centre (Canada)	Case-only
